# Study protocol for POSITIF, a randomised multicentre feasibility trial of a brief cognitive-behavioural intervention plus information versus information alone for the treatment of post-stroke fatigue

**DOI:** 10.1186/s40814-020-00622-0

**Published:** 2020-06-15

**Authors:** David C. Gillespie, Mark Barber, Marian C. Brady, Alan Carson, Trudie Chalder, Yvonne Chun, Vera Cvoro, Martin Dennis, Maree Hackett, Euan Haig, Allan House, Steff Lewis, Richard Parker, Fiona Wee, Simiao Wu, Gillian Mead

**Affiliations:** 1 Department of Clinical Neurosciences, Royal Infirmary of Edinburgh, Edinburgh, UK; 2 NHS Lanarkshire, Monklands Hospital, Coatbridge, UK; 3 Nursing, Midwifery and Allied Health Professions Research Unit, Glasgow Caledonian University, Glasgow, UK; 4 Department of Psychological Medicine, King’s College London, London, UK; 5 Centre for Clinical Brain Sciences, University of Edinburgh, Edinburgh, UK; 6 The George Institute for Global Health, Faculty of Medicine, University of New South Wales, Sydney, Australia; 7 Independent Consultant, Edinburgh, UK; 8 Faculty of Medicine and Health, University of Leeds, Leeds, UK; 9 Edinburgh Clinical Trials Unit (ECTU), University of Edinburgh, Edinburgh, UK; 10 Department of Neurology, West China Hospital, Chengdu, China

**Keywords:** Stroke, Fatigue, Physical activity, Rehabilitation, Psychological, Cognitive behavioural approach, Telephone, Clinical trial

## Abstract

**Background:**

Approximately, half of stroke survivors experience fatigue. Fatigue may persist for many months and interferes with participation in everyday activities and has a negative impact on social and family relationships, return to work, and quality of life. Fatigue is among the top 10 priorities for ‘Life after Stroke’ research for stroke survivors, carers, and clinicians. We previously developed and tested in a small uncontrolled pilot study a manualised, clinical psychologist-delivered, face-to-face intervention, informed by cognitive behavioural therapy (CBT). We then adapted it for delivery by trained therapists via telephone. We now aim to test the feasibility of this approach in a parallel group, randomised controlled feasibility trial (**P**ost **S**troke **I**ntervention **T**rial **I**n **F**atigue, POSITIF).

**Methods/design:**

POSITIF aims to recruit 75 stroke survivors between 3 months and 2 years post-stroke who would like treatment for their fatigue. Eligible consenting stroke survivors will be randomised to either a 7-session manualised telephone-delivered intervention based on CBT principles plus information about fatigue, or information only. The aims of the intervention are to (i) provide an explanation for post-stroke fatigue, in particular that it is potentially reversible (an educational approach), (ii) encourage participants to overcome the fear of taking physical activity and challenge negative thinking (a cognitive approach) and (iii) promote a balance between daily activities, rest and sleep and then gradually increase levels of physical activity (a behavioural approach). Fatigue, mood, quality of life, return to work and putative mediators will be assessed at baseline (just before randomisation), at the end of treatment and 6 months after randomisation. POSITIF will determine the feasibility of recruitment, adherence to the intervention and the resources required to deliver the intervention in a larger trial.

**Discussion:**

The POSITIF feasibility trial will recruit until 31 January 2020. Data will inform the utility and design of a future adequately powered randomised controlled trial.

**Trial registration:**

ClinicalTrials.gov, NCT03551327. Registered on 11 June 2018.

## Background

Approximately, 130,000 people have a stroke each year in the UK [1, 2]. Of these, almost a half will experience post-stroke fatigue [3]. Fatigue can be defined as a subjective feeling of lack of energy, weariness and aversion to effort [4] and in many cases, becomes a chronic symptom that has an adverse effect on a person’s ability to manage everyday activities, socialise and maintain intimate relationships and to return to paid employment [5, 6]. The self-reported quality of life of fatigued stroke survivors is often very low [7]. It is therefore not surprising that the need to find effective treatments for fatigue was amongst the top 10 priorities for ‘Life after Stroke’ research shared by stroke survivors, carers, clinicians and researchers [8].

**Fig. 1:**
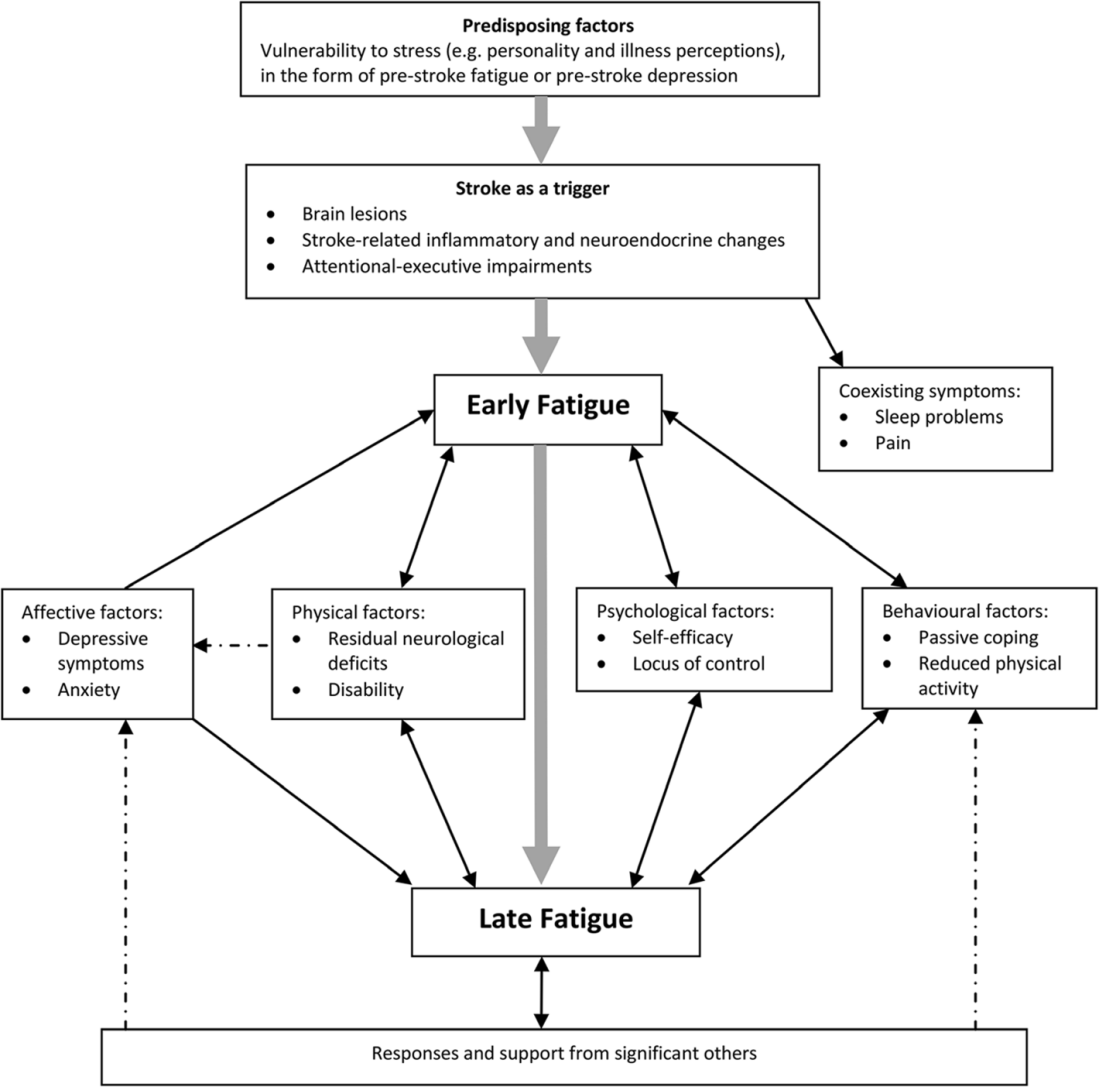
A conceptual model of post-stroke a fatigue (unidirectional arrows indicating an assumed causal direction and bidirectional arrows indicating an unknown direction of association. Dotted arrows indicate potential interactions between factors) from Stroke re-published with permission

The search for effective treatments for post-stroke fatigue has been challenging. There has been little success in identifying biological causes. For example, there is no clear association between fatigue and the severity of stroke or stroke lesion location [9]. Perhaps not surprisingly, biological treatments have so far been shown to be ineffective at alleviating fatigue in this population [10]. Therefore, in order to identify intervention targets in a broader context of stroke illness, we conducted a systematic review to explore the correlates of post-stroke fatigue [11]. Drawing on this evidence and our qualitative study of the experiences of individuals with post-stroke fatigue [12], we developed a stroke-specific model of fatigue, which proposed that depressive symptoms, anxiety, low self-efficacy, passive coping, reduced physical activity, sleep problems and inadequate social support are all important factors in the development and/or maintenance of fatigue [13]. These associations are consistent with findings from a qualitative study in which patients with post-stroke fatigue reported that rehabilitation and good sleep improved fatigue symptoms [12]. Figure 1 presents a conceptual model of post-stroke fatigue based on our work.

The psychological, behavioural and environmental factors outlined above are consistent with cognitive behavioural models for functional neurological symptoms (e.g. [14]), where these factors are interconnected and may cause or maintain fatigue symptoms, and our earlier work treating fatigue in the population [14, 15] and cancer-related fatigue [16]. The premise of cognitive behavioural therapy (CBT) is that addressing unhelpful thoughts and behaviours can change how people feel physically and emotionally [17]. We hypothesised [13] that increasing physical activity would be a starting point to break this ‘vicious cycle’ of post-stroke fatigue, because increasing activity levels would challenge cognitive barriers to activity (e.g. ‘This discomfort when I move is a sign I should take it easy’). The experience of being more active would then improve patients’ self-efficacy in taking physical activity and thus reduce fatigue and improve mood [18]. There is an association between post-stroke fatigue and inactivity [19], and, therefore, increasing activity levels after stroke is a key component of the intervention.

To test these ideas, we created a brief manualised intervention with input from stroke survivors and stroke clinicians [20]. The intervention aimed firstly to provide individuals with an explanation of post-stroke fatigue based on the psychological factors identified following systematic review of the literature, and to explain that the impact of post-stroke fatigue is reversible. Secondly, it encouraged fatigued stroke survivors to overcome any fears they might have about taking physical activity, specifically to challenge negative thoughts about their fatigue (e.g. ‘There’s nothing I can do about this’), that is to say a cognitive approach. Thirdly, the intervention promoted a balance between daily activities, rest and sleep, aiming for individuals to increase in increments their level of physical activity using diary monitoring and activity scheduling, in other words, a behavioural approach [14]. At the development phase, the intervention was delivered in an uncontrolled pilot study to 12 participants with post-stroke fatigue by a clinical psychologist with a special interest in stroke, to determine preliminary acceptability and feasibility [20]. The intervention comprised a participant handbook that included forms for diary keeping, six face-to-face treatment sessions and one follow-up telephone-delivered review (‘booster’) session. Fatigue levels were lower at the end of the study than at baseline for the eight individuals who completed treatment, and all participants reported favourable opinions of the intervention [20].

Following publication of the findings from this small observational trial, the trial intervention handbooks were edited taking into account participant feedback. We added new information about stroke survivors’ experiences of fatigue following a workshop about post-stroke fatigue at the Stroke Association UK Stroke Assembly (a conference for stroke survivors) in July 2017. At this point, a decision was made that for the next stage of intervention development, the Post Stroke Intervention Trial In Fatigue (POSITIF) would be delivered by trained nurses or other allied health care professionals (AHPs) or psychology graduates with training in CBT rather than clinical psychologists; the latter are too few in number to deliver this intervention across a national health care system, but nurses and other AHPs are core members of most stroke care teams, and are potentially a more cost effective option.

For pragmatic reasons, it was decided that the intervention would have a better chance of being accepted by clinical services if it could be offered to participants via telephone rather than face-to-face. This is because stroke services would struggle to offer treatments, even ones that are clinically effective, if the resources required to deliver them were too great. We were also mindful of the fact that many stroke survivors, particularly those with fatigue, struggle to get to hospitals and clinics so telephone delivery might offer a convenient way to receive a psychological treatment.

A telephone approach is justified as there is evidence from the literature that telephone-based delivery of CBT is feasible and effective. For example, a meta-analysis of 8 studies recruiting 658 participants with a range of physical problems including stroke revealed that telephone counselling, delivered by therapists or psychologists, resulted in significant improvements in coping skills and strategies, community integration and reduction in depression [21]. In participants with traumatic brain injury, seven scheduled telephone sessions over 9 months designed to elicit current concerns, provide information and facilitate problem solving in domains relevant to traumatic brain injury recovery that were associated with lower depression scores compared with usual care [22]. Our previous work in chronic fatigue syndrome had suggested that telephone treatment was acceptable to patients in a small randomised controlled trial [23].

The aim of this trial is to assess the feasibility of our trial methods.

## Methods

### Study registration

The study design was approved by the East of Scotland Research Ethics Committee (30 January 2018). The trial was registered with the U.S. National Institutes of Health on ClinicalTrials.gov (11 June 2018) with trial identification NCT03551327. Recruitment commenced in January 2019 and is currently ongoing.

### Trial design

The study is a UK-based, multi-site, randomised controlled feasibility trial including participants with post-stroke fatigue with broad entry criteria and follow-up to ascertain outcomes at 6 months after randomisation. The primary aim of the POSITIF feasibility trial is to ascertain recruitment rates, participant adherence to the intervention and completeness of data capture during intervention delivery and at follow-up. At the time of submission to ClinicalTrials.gov, our intention was to move seamlessly from a feasibility trial to an efficacy trial without stopping and analysing data. However, our application for further funding was not successful and so a decision was made to analyse and report data from the feasibility trial as described in this paper, in line with our pre-specified criteria for moving to the main phase of the trial.

**Fig. 2:**
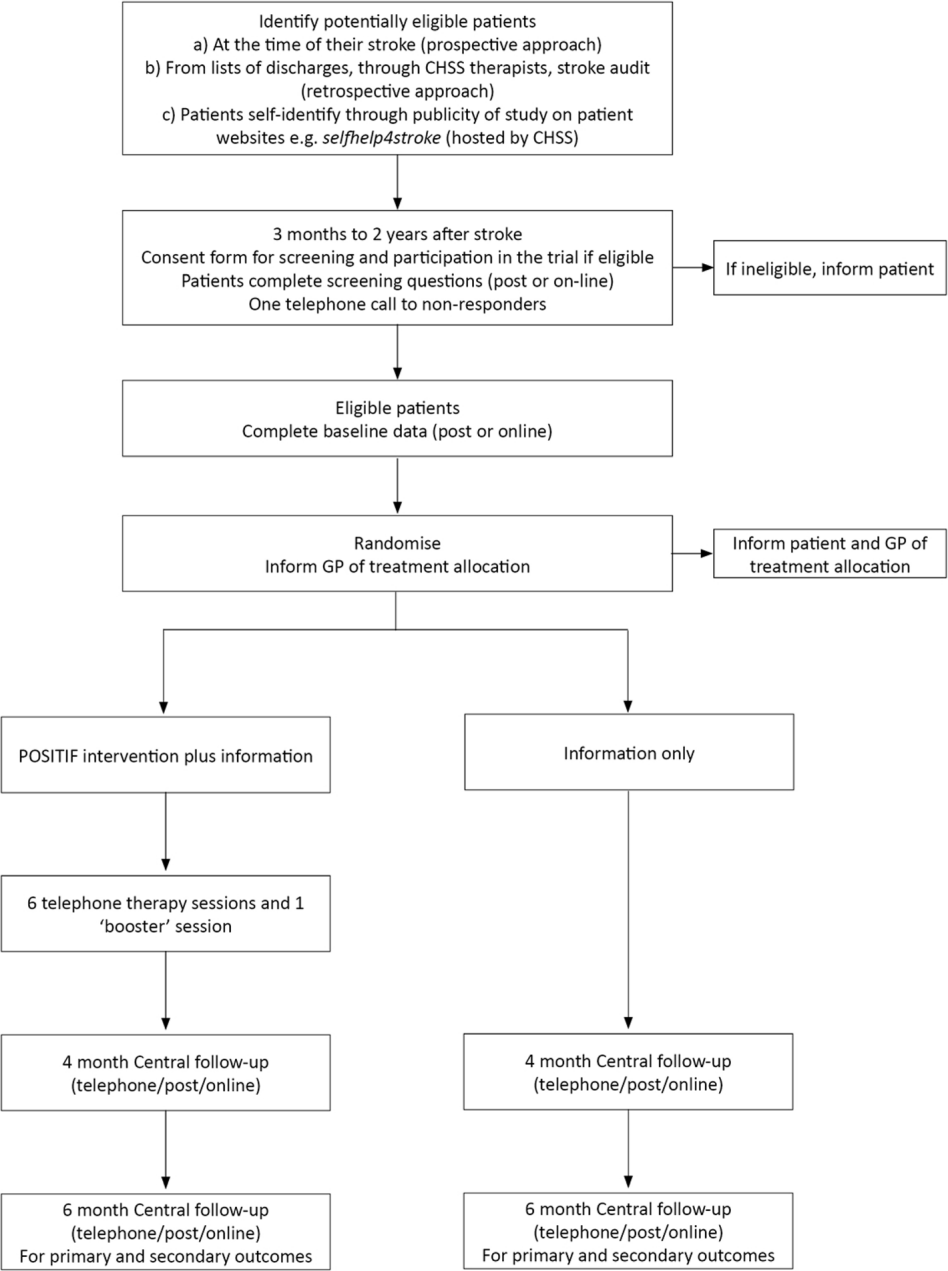
The POSITIF recruitment process

The expected flow of participants through the trial is shown in Fig. 2.

### Sample size

The recruitment period is 14 months, and we aim to recruit at least 75 participants in total from three hospital sites and online self-identification. This number is consistent with recommended sample sizes for pilot randomised controlled trials (RCTs) [24], 60–100 participants generally being required to estimate event rates in a single group. We will monitor recruitment rates closely so that any issues or barriers to recruitment can be identified and resolved.

### Participants

We aim to recruit participants who meet the eligibility criteria (see below). A three-pronged approach to recruitment, namely, retrospective, prospective and self-identification will be used.

For retrospective recruitment, each site will identify potentially eligible participants by screening stroke registers, audit databases, patient hospital discharge summaries and The Scottish Health Research Register (SHARE) and through nurses and support workers who see patients after discharge from hospital. In Scotland, nurses from the charity Chest Heart and Stroke Scotland (CHSS) visit most patients after discharge from hospital. The retrospective approach will be used until the ‘pool’ of potential participants who have had a stroke within the previous 2 years has been reached. Individuals identified as being potentially eligible will be contacted by letter or in person by a member of the clinical team (including, but not limited to, the lead clinician for the service, the individual’s hospital consultant or charge nurse) with an invitation to participate in the screening process and the trial if eligible; a participant information sheet and a consent form will be provided. If no response is received from the letter of invitation within 2 weeks, the local team will telephone the individual to ensure that the information was received and answer any questions they might have. Screening questionnaires and consent forms will be completed by participants at home and returned to the research team by post or by email.

For prospective recruitment, participants will be identified shortly after their hospital admission for stroke, at outpatient stroke clinics or soon after discharge from hospital by the stroke nurses who visit patients in the community (generally three weeks following discharge). The initial approach to the patient will be in person by a member of the clinical team. Consent will be obtained for subsequent screening 3 months after stroke onset and for participation in the trial if eligible. If a patient is ‘missed’ whilst in hospital (e.g. due to a very short hospital stay), then they will be contacted as for the retrospectively recruited participants. If a patient consents but does not return the screening questionnaires, a telephone call will be made to find out if they wish to take part.

Finally, individuals will be able to identify themselves as potential feasibility trial participants from the web links of the UK’s two main stroke charities, CHSS (*Selfhelp4stroke*, a self-management resource for anyone who has had a stroke, and *Stroke4carers*, an information and signposting portal for the carers and families of stroke survivors) and The Stroke Association (*My Stroke Guide*, a website providing resources and information to people affected by stroke). Those who are recruited in this way will participate in screening as described above.

### Inclusion criteria

- Age ≥ 18 years
- Stroke (ischaemic or haemorrhagic, first or recurrent, and including subarachnoid haemorrhage) between 3 months and 2 years previously, based on clinical diagnosis and compatible imaging
- Capacity to provide informed consent
- Not living in a nursing home at time of consent
- Medically stable
- Answers ‘Yes’ to both of the following questions from the Greater Manchester Stroke Assessment Tool (GM-SAT) [25] about fatigue: ‘Do you feel tired all the time or get tired very quickly since your stroke’? and ‘Would you like additional help and support for this’?

### Exclusion criteria

- Unlikely to be available for follow-up for the next 6 months, e.g. no fixed home address
- Life-threatening illness (e.g. advanced cancer or advanced heart failure) that would make survival for 6 months unlikely
- Aphasia or cognitive impairment severe enough to prevent participation in the intervention. To assess this, participants will self-report their language and cognition from the relevant domains of the Short Stroke Impact Scale (SF-SIS) [26] (‘In the past week, how difficult was it for you to think quickly’? and ‘In the past week, how difficult was it to understand what was being said to you in a conversation’?). Those who respond ‘very difficult’ or ‘could not do at all’ to either question, or those whose communication impairment means that they are unable to respond to such questions at all, will be excluded
- Actively suicidal, requiring in-patient treatment for depression or experiencing depression-related cognitive impairment
- High anxiety as part of a post-traumatic stress disorder (PTSD) syndrome or panic disorder
- Previously enrolled in this trial
- Enrolled in another trial of psychological therapy
- Enrolled in another trial of physical activity
- Inability to understand spoken and/or written English

If a participant scores ≥ 15 (the threshold for moderately severe depression) or scores 1, 2 or 3 on the suicidal item on the Patient Health Questionnaire 9-item (PHQ-9) [27], they will be telephoned by a member of the research team to ascertain the individual’s level of risk. We will write to the participant and their General Practitioner (GP). The GP will be advised of the score and what this means, and that we suggest further investigation and treatment. The same procedure—writing to participant and GP—will take place if a participant scores ≥ 15 on the Generalised Anxiety Disorder 7-item (GAD-7) [28] (the usual threshold for severe anxiety). We will not inform participants of their scores but we will inform them that screening has indicated possible problems with their mood or anxiety, and that we strongly encourage them to discuss this with their GP. Individuals will be excluded only if they are actively suicidal, requiring in-patient treatment or have depression-related cognitive impairment or if the individual has a PTSD syndrome or panic disorder (see above). In such situations, specific intervention from psychiatry or psychology would be needed before joining the POSITIF trial.

### Co-enrolment

Inclusion in another research study, including another randomised controlled trial, will not automatically exclude an individual from participating in POSITIF. As long as inclusion in the other study would not confound the results of POSITIF, co-enrolment will be permissible.

However, if someone has already been enrolled into a trial of a psychological therapy, they cannot be enrolled into POSITIF. If a participant is enrolled into POSITIF and is still being follow-up, they may not subsequently be enrolled into a trial of psychological therapy. If an individual has been recruited to a trial of a physical activity intervention, we will not allow them to take part in POSITIF.

When considering co-enrolment, we will be mindful of the potential burden upon participants, their families and research staff.

### Consent to participate

Eligible participants will be given a Patient Information Sheet (PIS) that explains what is involved in the study and an Informed Consent Form (ICF). If the potential participant requires additional information, they can contact the POSITIF trial team in the Edinburgh Clinical Trials Unit (ECTU) or the local investigator. We have developed ‘easy access’ materials (PIS, ICF and participant handbook) to support the participation of people with communication difficulties with input from people with aphasia.

Participants will receive the PIS/ICF differently depending on how they are identified:

- Participants who are identified retrospectively will be sent an ‘invitation pack’ which includes an invitation cover letter, PIS, ICF and screening form
- Participants who are identified prospectively will be either given the PIS only (if an in-patient and stroke occurred < 3 months ago) and the ICF and screening form will be sent later, or they may be given/sent the invitation pack as described above if the stroke occurred ≥ 3 months ago
- Participants who self-refer will either give consent on-line (via a secure webpage) or they will be sent an invitation pack as described above (the participant will be able to choose their preference). Participants who consent on-line will be sent a paper copy of the PIS for their records.

### Screening

There will be 6 screening items:

- Two simple screening questions for fatigue [25]: (participants must answer ‘yes’ to both to be eligible): ‘Do you feel tired all the time or get tired very quickly since your stroke?; ‘Would you like additional help and support for this?’
- Patient Health Questionnaire-9 (PHQ 9) [27]. The scores range from 0–27, with a higher score representing more severe depression (0–4 none, 5–9 mild, 10–14 moderate, 15–19 moderately severe, 20–27 severe)
- Generalised anxiety disorder 7 (GAD-7) [28]. The score ranges from 0–21 with a higher score representing more severe anxiety (5, 10 and 15 are the thresholds for mild, moderate and severe anxiety, respectively)
- ‘In the past week, how difficult was it for you to think quickly?’ (cognitive item from the Short Stroke Impact Scale, SF-SIS) [26]
- ‘In the past week, how difficult was it to understand what was being said to you in a conversation?’ (language item from the SF-SIS) [26]
- Besides your stroke, do you have any other serious or life-threatening illnesses?

In addition to the screening items outlined above, information on stroke subtype will be obtained. We will assess this by asking the question ‘Was your stroke due to a bleed or a blood clot?’ We did consider extracting information about stroke type from all participants’ medical case notes, but whilst this would be possible for participants identified by local recruitment sites, it would not be so easy to obtain this information for participants who self-identify for the study. In this feasibility trial, we will assess the agreement between participant report and case note diagnosis for participants recruited locally. This will enable us to estimate the extent of incorrect classification. We will also collect self-reported Modified Rankin Score (simple question version) [29] to obtain information on level of dependence, and we will ask individuals to list their medications and then answer the following question ‘Do you suffer from any of the following illnesses: cancer, heart failure, rheumatoid arthritis, Parkinson’s disease, multiple sclerosis?’ because all of these illnesses can cause fatigue [14, 15].

Those eligible will be asked to complete baseline data and will be randomised through the trial database. If the participant completes the screening questionnaires, and is found to be eligible for randomisation, they will be sent a baseline data collection form for completion (either by email or by post), and will then be randomised.

We anticipate that some participants may change their mind after being found to be eligible to participate. If we do not receive the baseline data, the participant will be telephoned. If the baseline data are not returned even after a telephone call, we will categorise the participant as having consented and been found to be eligible, but not randomised.

### Withdrawal of study participants

Participants may consent, be eligible and then change their minds at a later time. The participant’s doctor may advise them to stop participating in the intervention. We will record how often each of these scenarios occur. We will still follow-up the participant per protocol and collect 6 months follow-up data for the primary analyses. However, if a participant chooses to withdraw completely from the trial and not participate in follow-up, we will retain the data collected on that participant up to that point.

If a participant loses capacity during the trial, no further follow-up will be obtained but we will retain data already collected.

This intervention is low risk, and we do not anticipate major problems. However, should a participant develop any contraindication to participation during the intervention, for example due to severe deterioration in mental state, they will stop participating in the intervention but will continue to be followed up. If the contraindication resolves within 4 weeks, the participant will be allowed to restart the intervention and complete the six sessions and the booster phone call.

### Randomisation

Each participant’s screening and baseline data will be entered into a computerised central randomisation service by means of a secure 24/7 web interface. After the computer program has checked data for completeness and consistency, it will allocate the participant a unique study identification number and assign them to either the intervention or the control arm of the study. The system applies a minimisation program to achieve balance for three factors, namely, (i) time since stroke (as fatigue tends to improve over time; < 1 year versus ≥ 1 year) [3], (ii) sex (since fatigue tends to be more common in women) [30] and (iii) depression score at baseline (since those with more depressive symptoms may have more severe fatigue and so respond differently to the intervention) [11]. Minimisation on anxiety and fatigue will not be required because depression, anxiety and fatigue tend to be highly correlated [11].

The randomisation record will be stored within the trial database. Following randomisation to either the intervention or control arm, a letter will be sent to the participant’s GP to inform them of their patient’s enrolment in the trial with a copy of the signed participant consent form. The participant will be informed by telephone, email or post about the randomisation outcome. The intervention will commence within 2 weeks of randomisation, depending on the availability of therapist and participant.

### Intervention

This is a pragmatic trial. As stroke psychologists are scarce in the United Kingdom National Health Service (NHS) [31], it was decided that nurses or AHPs would be trained to deliver the intervention. The nurse/AHP therapists will receive standardised training from an experienced stroke clinical psychologist (DG) and fatigue expert and cognitive behavioural psychotherapist (TC) in how to deliver the intervention, and how to record the content of each session using a checklist. The 1-day training will comprise an overview of the literature on post-stroke fatigue as well as an introduction to the principles and practice of CBT, including role play and group discussion; reading materials for self-study will be also provided.

The delivery of the intervention will be by telephone. Trial materials (including written information about fatigue, a participant handbook, participant diary and follow-up questionnaires) have been made available on a trial website (www.ed.ac.uk/usher/edinburgh-clinical-trials/our-studies/all-current-studies/positif/the-positif-trial).

The intervention will be tailored towards the specific needs of participants, including their existing activity levels. For example, after the first session, the participant completes diaries to record activity and sleep. Then, based on those diaries, goals related to increasing activity and improving sleep are negotiated. The content of goals will be individualised and inevitably vary between participants. The participant handbook includes information sheets about how pain, medication and other medical conditions may be related to fatigue; participants will be sign-posted to the particular information relevant to them.

The intervention comprises six sessions, each separated by 2 weeks. In the intervals between sessions, it is suggested that participants work on their chosen goals. At the final session, participants reflect on any gains made, how they were achieved, discuss potential set-backs and make a plan to maintain and/or build on any behavioural changes they have made which may be influencing levels of fatigue. There is a review phone call two to four weeks after the sixth session, to check on progress and to offer ongoing encouragement. An outline of the content and main focus of each session is provided in Table 1.

**Table 1:** Content of telephone-delivered cognitive behavioural therapy sessions

Session number	Treatment outline
1	**Engagement and preparation** ­ Discuss the patient’s experience of post-stroke fatigue ­ Explain symptoms and potential mechanisms of post-stroke fatigue ­ Emphasise that maintaining factors are potentially reversible ­ Explain how to use a diary to monitor daily activities, rest and sleep
2	**Balancing daily activities, rest and sleep** ­ Review diaries the patient has been keeping to determine current levels of activity, rest and sleep ­ Discuss strategies to improve sleep patterns ­ Set SMART goals to increase daily activities and improve sleep ­ Agree on an initial plan to balance activity levels, rest and sleep
3	**Increasing daily activities in graded increments** ­ Review the patient’s diary and discuss progress with the initial plan ­ Discuss new goals to be achieved in the coming weeks (including decreasing the amount of rest) ­ Agree on a weekly plan to work towards new goals
4	**Improving emotions and thoughts** ­ Discuss the ‘3-area model’ to explain the links between thoughts, emotions and behaviour ­ Discuss the unhelpful thoughts and emotions that might occur in response to fatigue ­ Introduce thought challenging sheets
5	**Dealing with difficulties in making progress** ­ Identify common ‘blocks’ and setbacks in making progress ­ Discuss any problems the patient has experienced and agree with the patient solutions (patient taking active role)
6	**Preparing for the future** ­ Check patient’s understanding of the intervention and discuss their progress ­ Encourage the patient to suggest new future targets and a plan for working towards them ­ Ask patient to fill out treatment evaluation forms
Booster (4 mo after starting intervention)	**Review of overall progress** ­ Evaluate the patient’s progress since session 6 ­ Help the patient solve any outstanding problems ­ Review the patient’s understanding of treatment rationale and skills ­ Discuss further targets and plans

All sessions will be audio recorded on encrypted voice dictation devices. Nurse/AHP therapists will receive fortnightly telephone supervision for the duration of the study (approximately 30 min per fortnight); the supervising psychologist (DG) will have the opportunity to listen to audio recordings before supervision sessions. Table 2 provides an overview of the rationale for the study, as well as study materials and procedures.

**Table 2:** Overview of intervention rationale, materials and procedures

Brief name of intervention	**P**ost **S**troke **I**ntervention **T**rial **I**n **F**atigue (The POSITIF Trial)
**Why (rationale for treatment)**	Post-stroke fatigue is common, experienced by approximately half of all stroke survivors. It has a negative impact on a range of important life domains. A systematic review of the literature found that psychological factors, namely, depression, anxiety, low self-efficacy, passive coping, reduced physical activity, sleep problems and low levels of social support are implicated in the development or maintenance of fatigue following stroke. This evidence suggests that cognitive behavioural treatment methods, which target individuals’ thoughts, behaviours and feelings, and have been used to treat fatigue in other health conditions, could be effective in the treatment of post-stroke fatigue.
**What (materials)**	POSITIF is a manualised cognitive behaviourally informed treatment that targets the factors that have been associated with post-stroke fatigue in the literature. Individuals will receive a participant manual that includes written information about post-stroke fatigue, as well as activity and sleep diaries and worksheets for goal setting and thought challenging. Before POSITIF, the materials were provided to 12 stroke survivors in a small uncontrolled pilot study and edited to take account of participant and clinician feedback (see Table 1).
**What (procedures)**	Information will be provided to participants about post-stroke fatigue and individuals will be given an opportunity to discuss their ‘model’ of fatigue, i.e. why they believe they experience it. Any misconceptions about fatigue will be corrected. Activity diaries and sleep diaries will be completed by participants throughout the intervention and sent to the therapist (by post); these will form the basis for a tailored approach designed to promote a balance between daily activities, rest and sleep, the aims being to gradually increase levels of physical activity, and to avoid ‘boom and bust’ activity patterns. Therapists will identify participant beliefs about fatigue and help participants to challenge negative thinking, encouraging them overcome any fears about undertaking physical activity (see Table 1). The comparator group will receive written information about fatigue only, in the form of a leaflet (available at www.strokeassociation/dudfuhfud.com).
**Who (profession, expertise, specific training, etc)**	The intervention is to be delivered by nurses or Allied Health Professionals (AHPs). These therapists will be individuals with clinical experience of stroke, but no prior training in Cognitive Behavioural Therapy (CBT). They will be representative of the nurses who work with stroke survivors in community stroke settings. Therapists will receive a one-day training that comprises an overview of the literature on post-stroke fatigue, an introduction to the principles and practice of CBT, and information on how to deliver the intervention, including how to record the content of sessions. A stroke clinical psychologist and a cognitive behavioural psychotherapist will deliver the training. Brief role plays and group discussions will be included; reading materials, including journal articles will be provided to trial therapists for self-study. Nurse/AHP therapists will receive fortnightly telephone supervision (30-minutes duration) from the stroke clinical psychologist who delivers the training.
**How (modes of delivery)**	POSITIF sessions will be telephone-delivered. Phone calls will be made at times convenient to participants. Therapists will try to call participants at least two or three times before a session is classed as ‘missed’, as would happen in clinical practice. Participants will be required to have their written manuals in front of them during the calls so that therapists can direct them to particular worksheets and other materials.
**Where (infrastructure and relevant features)**	Participants will receive the telephone sessions in their own homes. They will receive the participant manuals by post.
**When and how much (number of sessions, duration, intensity, dose)**	The intervention comprises six sessions, one every two weeks. Sessions will be up to 60 minutes in duration. In the intervals between sessions, participants will work on their chosen goals. A review ‘booster’ telephone session will take place two to four weeks after the sixth session.
**Tailoring (personalisation)**	Goals will be individualised for each patient to take account of their baseline level of activity and sleep patterns, their physical health, levels of fatigue and their interests and aspirations.
**Modifications (from existing or initial protocol)**	Any modifications that are required in the course of the intervention will be recorded.
**How well (planned adherence)**	Adherence to the intervention will be determined as number of sessions each participant receives.

### Comparator

Careful consideration was given to the nature of the control intervention. As there is no effective intervention for post-stroke fatigue that is routinely available in clinical practice, it is not possible to compare the active intervention in POSITIF with another active intervention. A ‘placebo’ control intervention such as relaxation was considered, but would have required considerable additional resources to administer, and may have been less acceptable to participants [32]. A wait list design was also considered in which no intervention would be provided during the experimental treatment period, with active treatment offered after the final follow-up assessment, but this would also have needed additional resources. The participant handbook could have been provided to control participants at the end of the post-treatment assessment, but it was designed to be used with therapist input and could not be easily used by individuals without support and guidance. Finally, the control could have been ‘usual care’, but there were concerns that participants might decline the invitation to participate if there was a 50% probability of receiving nothing additional to routine care. Therefore, it was decided to provide control participants with information about fatigue. Although information about post-stroke fatigue is easily available (e.g. from charity websites), our clinical experience is that patients are not usually signposted to it. A Cochrane review of information provision after stroke reported that information alone has a very small, probably not clinically significant effect on depression, though there are no data on the effect of information provision on fatigue [33]. A copy of the information leaflet, given immediately following randomisation, can be found at https://www.stroke.org.uk/sites/default/files/fatigue_after_stroke.pdf.

Table 3 provides a summary of the timing of treatment telephone calls and feasibility study assessments.

**Table 3:** Summary of study assessments and treatment telephone calls

	Study period										
	Enrolment	Allocation	Post-allocation							Close-out	
Timepoint (time in weeks)	−t1	0	1	3	5	7	9	11	14 (+/−1)^*^	16	26
Enrolment											
Consent and screening	X										
Randomisation	X										
Allocation		X									
Intervention											
Telephone call 1			X								
Telephone call 2				X							
Telephone call 3					X						
Telephone call 4						X					
Telephone call 5							X				
Telephone call 6								X			
Booster telephone call									X		
Assessments											
Baseline assessments											
Two fatigue screening questions PHQ-9 GAD-7 SF-SIS cognitive item SF-SIS language item Screening questions about serious illness		X									
4 months follow-up assessments											
FAS PHQ-9 GAD-7 CBRQ EQ-5D-5 L										X	
6 months follow-up assessments											
FAS PHQ-9 GAD-7 CBRQ SF-SIS EQ-5D-5 L Anxiolytics (Y/N) Hours working Health costs											X

*The booster treatment telephone call can take place at 13, 14 or 15 weeks post-randomisation

*CBRQ* Cognitive and Behavioural Responses Questionnaire, *EQ-5D-5 L* EuroQoL 5-dimension 5-level, *FAS* Fatigue Assessment Scale, *GAD-7* Generalized Anxiety Disorder Assessment 7-item, *PHQ-9* Patient Health Questionnaire 9-item, *SF-SIS* Short Form Stroke Impact Scale

### Feasibility outcomes

The following data will help to inform the design of a future main efficacy trial:

#### Recruitment

- The feasibility of individuals referring themselves for fatigue screening via links to patient websites including CHSS and The Stroke Association (number and rate)
- The feasibility of identifying stroke survivors through local sites (number and rate) and the proportion who agree to fatigue screening
- The number of stroke survivors who complete the screening questions, according to the method by which they were identified (by local sites or self-identification)
- The proportion of individuals who undergo fatigue screening who are eligible to participate
- The proportion of eligible patients who are randomised
- The recruitment rates by different methods and by different sites
- The feasibility of identifying and training nurses and AHPs to deliver the intervention

#### Adherence and retention

- The adherence rate and reasons for non-adherence (number of sessions participants receive)
- The fidelity of the intervention (i.e. therapist adherence to the manual)

#### Data completion and data variability

- The response and completion rates for postal and web-based questionnaires, and the proportion of participants requiring telephone calls to collect follow-up questionnaires
- Variability in our fatigue outcome measure

#### Resources

- The resources required to deliver the intervention

A Data Monitoring Committee (DMC) will be established, and a DMC charter will be written. In this feasibility trial, the Trial Management Group (the investigators) will provide oversight of the trial and will function as a Trial Steering Committee (TSC).

### Progression criteria

We will proceed to an efficacy trial if the following criteria are achieved in the feasibility trial:

- Recruit 75 participants in 1 year (from local sites and through self-referral, e.g. links with relevant websites)
- Follow-up (primary outcome in at least 90% of participants)
- Adherence to therapy: at least four of the six sessions attended

If these criteria are not met, the TMG will explore the reasons for this and may still proceed with an efficacy trial if the reasons are addressable, for example sick leave amongst therapists delivering the intervention.

### Potential future outcome measures for an efficacy trial

The following measures are not outcomes for this feasibility trial, but are potential measures for a future efficacy trial:

- The Fatigue Assessment Scale (FAS). The FAS is a 10-item self-report scale that is valid and reliable in stroke [34]. The scale measures mental and physical fatigue. A difference of four points is considered to represent a clinically relevant change on this scale [35].
- Self-reported mood assessed using PHQ-9 [27] and the GAD-7 [28]. We will enquire whether antidepressants or anxiolytics have been prescribed
- Self-reported fearful beliefs in relation to exercise, to determine if these are a mediator in the effect of CBT on post-stroke fatigue, measured using the fear avoidance questions from the Cognitive and Behavioural Responses Questionnaire (CBRQ) [36]. The decision to include the CBRQ was made after the trial was registered with the National Institutes of Health, as an amendment to our protocol
- Stroke specific quality of life including participant-reported social participation assessed using the Short Form of the Stroke Impact Scale (SF-SIS) [26]
- Quality of life adjusted life years (QALYs) assessed using the Euroqol 5D (5-level version) [37].
- The number of hours participants are working relative to before their stroke (participants asked to report average hours worked per week currently and immediately before stroke).
- We will collect data on health costs (visits to the GP, number of admissions to hospital, days in a care home, number of visits from social carers, cost of the therapist delivery time, cost of the supervision time from psychology/psychiatry). In a future efficacy trial, this would enable us to perform a health economic analysis which will tell us how much the intervention costs, whether there are savings in use of health/social care and what QALYs are associated with the intervention.

### Statistical analysis

We will report numbers and percentages for the tests of feasibility, without formal statistical testing.

For the proposed outcome measures for a future efficacy trial, in this feasibility study, we will present overall summaries across both treatment groups combined, such as mean and standard deviation, number and percentage and proportion of missing data. We will also test for differences between the randomised groups, but because this is a feasibility trial with a relatively small number of participants, we will refrain from drawing strong conclusions. A full statistical analysis plan will be written prior to database lock.

## Discussion

A high proportion of stroke survivors experiences fatigue. Post-stroke fatigue is negatively associated with physical, psychological and social functioning—particularly for those unfortunate enough to experience fatigue long term—and there is little existing evidence from RCTs of effective treatments. However, absence of evidence is not the same as evidence of absence; psychological/behavioural treatments for post-stroke fatigue have simply not yet been put to rigorous scientific test.

To address this gap, the feasibility stage of POSITIF aims to test the feasibility of a nurse/AHP provided telephone-delivered CBT-informed intervention for individuals who experience fatigue 3 months to 2 years following stroke. We have preliminary data from a previous pilot study that indicates that the content and style of the intervention is acceptable. Small improvements have been made to the intervention protocol in the light of participant and therapist pilot study feedback. The main changes from the published uncontrolled pilot study have been to move from face-to-face to telephone delivery of the intervention and to have nurses and AHPs rather than clinical psychologists deliver the intervention. These changes were made on clinical and pragmatic grounds. As far as mode of therapy delivery is concerned, there is published evidence for the efficacy of telephone-delivered CBT interventions in clinical health populations, including participants with neurological injury [21, 22] and chronic fatigue syndrome [23], though we do acknowledge that delivery by telephone may be less acceptable to individuals with communication impairment. Delivery of the intervention by nurses and AHPs rather than clinical psychologists is a more realistic proposition for health care systems such as the UK where clinical psychologists are in relatively short supply [31]. Therapists working in multidisciplinary IAPT (Improving Access to Psychological Therapies) teams could also deliver the intervention [38].

The intervention may have several advantages over traditional face-to-face delivered CBT. Because the intervention is delivered by telephone, participants will be spared the expense of time and effort to travel to clinic appointments. It is especially important to consider the demands made on fatigued individuals in interventions of this type, because potential participants might be reluctant to commit scarce energy reserves to such interventions; as noted in a recent Cochrane review, fatigue trials often have high dropout rates [10]. Though the intervention is manualised, there is scope within it to negotiate goals with each participant. Indeed, the emphasis is on empowering the individual at all stages of the intervention. It is hoped that this will enable individuals to make gains beyond the period of active treatment. The aim that participants become, in effect, their own therapists lies at the heart of CBT [17].

Improvements in fatigue are not the only outcome of interest. As well as mood outcomes (depression and anxiety), we will record individuals’ social participation, how likely they are to return to paid work and their overall quality of life. These are important outcomes, because one would hope that if the intervention delivered in the POSITIF trial improves functioning via activity scheduling, then participants’ level of fatigue will also improve. The importance of improvement in key life roles is well recognised by so-called ‘third wave’ cognitive behavioural therapies [39, 40] which help people connect with personal values and do what matters to them. We also expect that treatment effects may be mediated by changes in fearful beliefs. Mechanisms of change will be assessed in a future trial.

There are limitations and possible challenges. One is the possibility of loss to follow-up, particularly the attrition bias that would occur if the most fatigued participants dropped out of the study. Although the intervention is relatively brief (10 weeks duration, excluding the booster session), as with all cognitive behavioural therapies, it demands active involvement on the part of the participant, and home-based practice is expected following every treatment session. The key, we believe, is to make sure participants who sign up to the feasibility trial genuinely do want help to manage their fatigue and understand what is expected of them (without being off putting). Participant information sheets will make clear the active nature of the intervention. We will also perform a sensitivity analysis to compare the characteristics of individuals who complete the intervention with individuals who do not. We acknowledge that a limitation of this feasibility study is that intervention and control conditions are not matched for contact time, which may have a positive behavioural effect in itself. However, it would have been impractical within the constraints of funding to perform a three arm feasibility trial. A larger efficacy trial might match participants in intervention and control arms for therapist contact time, perhaps comparing individuals receiving POSITIF with individuals receiving an equivalent amount of non-directive discussion about the general impact of stroke on everyday life, such as the unstructured social contact delivered as the control arm in a trial of communication therapy for aphasia and dysarthria following stroke [41].

Fatigue is a serious complication of stroke, blighting the lives of the large number of individuals who experience it. The findings will determine whether a randomised trial involving larger numbers of participants with adequate statistical power is feasible and warranted.

## Data Availability

Requests for data sharing should be directed to ECTUdatashare@ed.ac.uk after the final analyses are complete.

## Abbreviations

AHP: Allied Health Professional
CBRQ: Cognitive and Behavioural Responses Questionnaire
CBT: Cognitive behavioural therapy
CHSS: Chest Heart and Stroke Scotland
DMC: Data Monitoring Committee
ECTU: Edinburgh Clinical Trials Unit
EQ-5D-5 L: EuroQoL 5-dimension 5-level
FAS: Fatigue Assessment Scale
GAD-7: Generalized Anxiety Disorder Assessment 7-item
GCP: Good Clinical Practice
GM-SAT: Greater Manchester Stroke Assessment Tool
GP: General Practitioner
HRQOL: Health-related quality of life
IAPT: Improving Access to Psychological Therapies
ICF: Informed Consent Form
NHS: National Health Service
PHQ-9: Patient Health Questionnaire 9-item
PIS: Patient Information Sheet
POSITIF: Post-stroke Intervention Trial in Fatigue
PTSD: Post-traumatic stress disorder
QALYs: Quality of life adjusted years
RCT: Randomised controlled trial
SF-SIS: Short Form Stroke Impact Scale
SHARE: Scottish Health Research
TSC: Trial Steering Committee
